# Safety and Immunogenicity of a Live Oral Recombinant Cholera Vaccine VA1.4: A Randomized, Placebo Controlled Trial in Healthy Adults in a Cholera Endemic Area in Kolkata, India

**DOI:** 10.1371/journal.pone.0099381

**Published:** 2014-07-01

**Authors:** Suman Kanungo, Bandana Sen, Thandavarayan Ramamurthy, Dipika Sur, Byomkesh Manna, Gururaja P. Pazhani, Goutam Chowdhury, Puja Jhunjhunwala, Ranjan K. Nandy, Hemanta Koley, Mihir Kumar Bhattacharya, Sanjay Gupta, Gaurav Goel, Bindu Dey, Thungapathra M, G. Balakrish Nair, Amit Ghosh, Dilip Mahalanabis

**Affiliations:** 1 Society for Applied Studies, Kolkata, West Bengal, India; 2 Department of Epidemiology, National Institute of Cholera & Enteric Diseases, Kolkata, West Bengal, India; 3 Department of Microbiology, National Institute of Cholera & Enteric Diseases, Kolkata, West Bengal, India; 4 Catalyst Clinical Services Pvt. Ltd., Delhi, India; 5 Department of Biotechnology, Ministry of Science and Technology, New Delhi, India; 6 Institute of Post Graduate Medicine and Research, Chandigarh, India; 7 Translational Health Science and Technology Institute, Gurgaon, Haryana, India; University of Ottawa, Canada

## Abstract

**Background:**

A live oral cholera vaccine VA 1.4 developed from a non-toxigenic *Vibrio cholerae* O1 El Tor strain using *ctxB* gene insertion was further developed into a clinical product following cGMP and was evaluated in a double-blind randomized placebo controlled parallel group two arm trial with allocation ratio of 1∶1 for safety and immunogenicity in men and women aged 18–60 years from Kolkata, India.

**Method:**

A lyophilized dose of 1.9×10^9^ CFU (n = 44) or a placebo (n = 43) reconstituted with a diluent was administered within 5 minutes of drinking 100 ml of a buffer solution made of sodium bicarbonate and ascorbic acid and a second dose on day 14.

**Result:**

The vaccine did not elicit any diarrhea related adverse events. Other adverse events were rare, mild and similar in two groups. One subject in the vaccine group excreted the vaccine strain on the second day after first dose. The proportion of participants who seroconverted (i.e. had 4-folds or higher rise in reciprocal titre) in the vaccine group were 65.9% (95% CI: 50.1%–79.5%) at both 7 days (i.e. after 1^st^ dose) and 21 days (i.e. after 2^nd^ dose). None of the placebo recipients seroconverted. Anti-cholera toxin antibody was detected in very few recipients of the vaccine.

**Conclusion:**

This study demonstrates that VA 1.4 at a single dose of 1.9×10^9^ is safe and immunogenic in adults from a cholera endemic region. No additional benefit after two doses was seen.

**Trial Registration:**

Clinical Trials Registry-India, National Institute of Medical Statistics (Indian Council of Medical Research) CTRI/2012/04/002582

## Introduction

Cholera causes rapid dehydration due to diarrhea and is caused by ingestion of toxigenic serogroups (O1 and O139) of *Vibrio cholerae*. Cholera occurs in endemic form in south and south-east Asia and in Africa. However, outbreaks occur in many countries. Recently, a growing numbers of major cholera outbreaks occurred and are causing concern. The global disease burden of cholera is estimated to be 3–5 million cases and 100,000–130,000 deaths per year which are believed to be underestimates by experts [1]. More recently in 2012 a total of about 2.5 million cases and over 3000 deaths were reported with a case-fatality rate of 1.2% [2]. WHO advises use of oral cholera vaccines to reduce mortality in cholera endemic areas where standard prevention and control measures including safe drinking water, improvement of hygiene and sanitation measures are difficult to implement.

Two types of killed oral cholera vaccine are currently available, (i) Dukoral based on formalin and heat-killed whole cells of *V. cholerae* O1 (classical and El Tor, Inaba and Ogawa) plus recombinant cholera toxin B subunit, given with a bicarbonate buffer, and (ii) Shanchol and mORCVAX, the two identical vaccines formulated by two different manufactures based on *V. cholerae* serogroups O1 and O139. These two vaccines require two doses for protection. The only licensed oral and live attenuated single-dose vaccine CVD 103-HgR is no longer produced.

A new oral candidate vaccine has been constructed from a non-toxigenic strain of *V. cholerae* O1 El Tor, Inaba, which is not only devoid of the cholera toxin (CT) virulence cassette but also is completely non-reactogenic in rabbit ileal loop assay [3]. The strain, however, has *tox*R and *tcp*A genes encoding the toxin regulation and toxin-coregulated pili, respectively. Through a series of manipulations, the *ctx*B gene of *V. cholerae*, responsible for the production of the ‘B’ subunit of the cholera toxin (CTB) was introduced into the cryptic hemolysin locus of the strain. The resulting strain, named vaccine attempt 1.3 (VA1.3), was found to be able to produce copious amounts of CTB. In the removable intestinal tie-adult rabbit diarrhea model (RITARD) model this strain was found to be non-reactogenic and provided full protection against the challenge doses of both *V. cholerae* O1, classical and El Tor. In a large human volunteer study with live oral cholera vaccine VA1.3 we have shown that seroconversion rate with this novel vaccine is excellent and the adverse effects are negligible [4]. Further, the vaccine strain was not excreted in the stool. This vaccine strain however has an ampicillin resistance marker and it was introduced for easy detection in the environment. Following advice from experts the developers of the vaccine have now deleted the marker and the modified strain is named VA1.4. VA1.4 was derived from the strain VA1.2 [3], which is identical to VA1.3, excepting that it carries only a single copy of the ampicillin resistance marker tagged to *ctxB* (*ctxB*.Amp) [3]. To achieve this, typical experimental procedures were carried out which involved diluting an overnight (18 hr) culture of VA 1.2 1000X, followed by the exposure of aliquots separately to a 15 W Philips Germicidal Lamp (emitting primarily at 254 nm) at a distance of 56 cm, delivering 2.1 J/m^2^/sec for 1 to 4 seconds; 50 µl aliquots from the cultures were then plated on LB-Agar plates and incubated overnight at 37°C. Subsequently these were then replica plated onto LB-Agar plates containing ampicillin at 50 mg/ml. Ampicillin sensitive colonies picked up from the master plates, were screened for the presence of functional *ctxB* and other attributes, after purification by several rounds of streaking. One colony which was identical to VA 1.3 in all tests was selected and designated VA1.4. This construct was then developed into a clinical product following cGMP by Shantha Biotechnics Pvt. Ltd., Hyderabad. We present here the results of a randomized, placebo controlled, double blind, parallel group two arm trial with allocation ratio of 1∶1 in adult volunteers to evaluate the safety and immunogenicity of the cholera vaccine VA 1.4 (an identical construct of VA 1.3).

## Methods

The protocol for this trial and supporting CONSORT check list are available as supporting information. See Checklist S1 and Protocol S1.

### 2.1 Study Objectives

The objectives are to evaluate the safety and immunogenicity of the investigational product (IP) i.e. live oral VA1.4 cholera vaccine (identical to VA1.3 except for absence of Ampicillin resistance marker) in adult volunteers in Kolkata, India, aged 18 years to 60 years.

#### 2.1.1 Study End Points

In this trial in adults we compared the live oral cholera vaccine and placebo recipients for: I occurrence of adverse events to show safety, e.g. diarrhea, vomiting, fever, abdominal pain or cramps, headache, loss of appetite, general ill feeling, rash; and II. Serum vibriocidal antibody response (≥4 fold geometric mean folds rise) to serogroup O1, to show immunogenicity.

#### 2.1.2 Regulatory Approvals

The study was approved by the scientific Advisory committee, Institutional Biosafety committee and Institutional Ethics Committee (IEC) of the National Institute of cholera and Enteric Diseases (Indian Council of Medical Research)) and the Ethics Review Committee of the Society for applied studies, Kolkata. The Investigational New Product (IP) has undergone toxicological studies by an accredited laboratory per schedule Y of the drugs and cosmetics Act and following Bio-safety norms of the genetically modified organisms as per the batch data specifications of the Review Committee on Genetic Manipulation (RCGM, Government of India). The RCGM reviewed the data and accorded approval to conduct the phase I/II clinical trials. This protocol was approved by the Drugs Controller General, India (DCGI) for human volunteer study using VA1.4 cholera vaccine as cGMP lot.

### 2.2 Participants

The study was conducted in the Clinical Trials Unit jointly administered by the Society for Applied Studies (SAS) and the National Institute of Cholera and Enteric Diseases (NICED) (Indian Council of Medical Research, ICMR), situated at the Infectious Diseases Hospital in Kolkata. The Clinical Trials Unit has a separate facility with independent entry and exit. It has space for 8–10 beds, a room for counseling, an attached clinical laboratory and a room for examination. There is a separate and dedicated toilet facility. Residents of Kolkata, India not residing in the areas covered by the cholera or other vaccine trials were recruited in the study. Healthy adults aged 18–60 years were recruited in the study between May and July 2012.

#### 2.2.1 Screening and Eligibility

Seven to 4 days prior to administration of the first dose of the vaccine screening procedures were conducted. Written informed consent was obtained prior to screening. Medical history was recorded in a pretested form and a physician examined them for any overt or underlying illness. Individuals who were pregnant, with abdominal pain, loss of appetite, nausea, general ill-feeling or vomiting in the preceding 24 hours; or diarrhea or history of anti-diarrheal or antibiotic use during the past two weeks; or history of diarrhea and abdominal pain lasting for more than 2 weeks during the past 6 months or any chronic illness were excluded. A routine blood test for hemoglobin, white blood cell count, platelets, kidney function tests and liver function tests were done. A blood sample for vibriocidal antibody titre and anti-toxin antibody titre was collected and stored. The vaccine was manufactured in August of 2011 with expiry in July 2012. The vaccine was administered between 4^th^ June and 11^th^ July 2012.

#### 2.2.2 Randomization & Blinding

A randomization list was prepared by a person not involved in the study. The randomization code was sequential numbers unique to each individual. The single dose bottles (two for each subject) containing either the vaccine or identical looking placebo were arranged according to the randomization code and serially numbered. The master randomization chart was prepared by a competent person not involved in the study. Randomization list was generated using NQuery Advisor Version 7.0 software with varying block lengths, with allocation ratio of 1∶1 for the vaccine and placebo.

### 2.3 Packaging, Coding and Administration

The vaccine was packaged in single dose vials in a lyophilized form. Similarly placebo was packaged in single dose vials in a lyophilized form. A live oral vaccine **VA1.4** was used. During administration of the agents, necessary numbers of vials with consecutive serial numbers (starting with Serial No. 1) were at hand. The agent to be administered was determined by the subject serial number which was the same as the serial number on the vial. The subject drank a buffer solution (bicarbonates 2.5 g and ascorbic acid 1.65 g in 100 ml). The freeze dried vaccine or placebo vials were reconstituted in 1 ml of diluents provided by the manufacturer and given directly into mouth to drink 5 minutes after drinking the buffer solution. The agent was administered with a disposable syringe (without needle). The subject did not receive any other medicine unless indicated and a Medication Form was filled. Study Doctor was to decide about the medication; however antibiotics were avoided if practical until they receive a single dose of Doxycycline or Azithromycin on day 21. The vaccine and placebo were stored at 4°−8°C in a designated refrigerator kept close to the study unit to meet the daily needs. Expected duration of follow-up of each subject was 21 days. Each subject was free to accept or reject the proposal to enroll himself/herself. Even after enrolment the subject was able to withdraw from the study at any time.

#### 2.3.1 Randomization Codes

Department of Biotechnology, Ministry of Science and Technology, Government of India held the code. The only persons in the field site with access to the codes were a designated member of the Independent Data Safety Monitoring Board (DSMB) and the independent onsite clinical monitor in Kolkata, who were handed the codes in sealed envelopes. They were allowed only to un-blind codes in the event of severe putative vaccine reactions. Otherwise the codes were not revealed until the end of the trial and follow-up and until the computerized data set to be used for the analysis of vaccine effectiveness has been frozen. After reviewing the data set and summary information the DSMB provided the investigators information of the code as groups A and B without disclosing the group identity. The investigators analyzed the data comparing the two groups and again submitted the results to DSMB who then revealed the identity of the groups.

#### 2.3.2 Identification of Source Data

The clinical record forms (CRF) had the identification number only. Name of the volunteers was on record only on the consent forms to be kept in a separate file. General instructions for filling the case report forms were provided with CRF’s.

#### 2.3.3 Interpretation of the results

(a) Acceptable level of adverse events mainly diarrhea, vomiting (i.e. ≤10%) was a necessary condition for a field trial.

(b) Similar rise in vibriocidal antibody titre [4] as after VA 1.3 was necessary for going into a field trial (same or above the lower 95% confidence interval i.e. 49%).

### 2.4 Study Procedure

The flow chart (Table 1) summarizes the procedures.

**Table 1:** Study Procedure.

Study Procedure	*Day -7 to -4	Day 0	Day 1 & 2		Day 3		Day 7	Day 14	Day 15–16	Day 17		Day 21
Informed Consent	X											
History and Physical Exam	X											
Screening	X											
Clinical Evaluation	X	X	X				X	X	X			X
Randomization		X										
Blood Draw - Lab Tests#	X											X
Blood Draw - Vibriocidal Assay	X						X					X
Administration of Study Agents- **Oral Cholera** **Vaccine VA1.4** or **Placebo**		X						X				
Stool/Rectal Swab - Shedding			X									
Solicited Symptoms (Reactogenicity symptoms)		X	X		X		X	X	X	X		X
Adverse Event Monitoring		X		X			X	X	X			X

**#** Routine Blood Test [Hb, TLC, DLC, Platelets].

KFT, LFT [Urea, Creatinine, Electrolyte (Na+, K+), Uric Acid] LFT [SGOT, SGPT, ALP, Total Proteins & Albumin, Total b l Bilirubin].

*Initial screening.

Four to 7 days prior to administering the first dose informed consent was obtained from the subject. All consenting subjects were assigned a unique study Identification (ID) number in consecutive sequence which means that all consenting subjects received an ID number regardless of whether they were medically eligible. Screening for eligibility criteria, history and physical examination were completed by the study physicians. Approximately 10 ml blood was obtained for laboratory evaluation including kidney function and liver function tests, and stored for baseline vibriocidal antibody titre and cholera toxin antibody titre assay.

On day zero, once the subject was included based on the laboratory reports and clinical assessment, a study serial number was assigned. The randomization of the IP and placebo was incorporated in the study serial number of the bottles of IP or placebo. The study agent was administered according to the assigned randomization number. The subject was observed in the clinic (day care unit) for 6 hours. They were observed for 2 more consecutive days with clinic visits with extended stay. Primary end points were the occurrence of adverse events and serum vibriocidal antibody response. Solicited adverse events were noted down on day 0,1 and 2 and on day 7 or 8. On day 7 blood sample (5 ml) was obtained for vibriocidal and anti-cholera toxin assay. On day 14 a second dose of study agent was given according to the assigned randomization number. The code for the volunteers on day 14 remained the same as that on day 0. The subject was asked to stay for 6 hours. Solicited symptoms and/or adverse events were recorded on day 15, 16 and 17. On day 21 subjects were evaluated clinically. Approximately 10 ml of blood was obtained for immunological response and safety assessment. Subjects were enrolled starting from June 4, 2012 till July 11, 2012. Follow-up was completed on August 1, 2012.

#### 2.4.1 Outcomes

The primary endpoints of the study were safety and immunogenicity. Safety was evaluated from the proportion of subjects exhibiting diarrheal adverse events during the study period whereas immunogenicity was evaluated from the proportion of subjects exhibiting 4-fold or greater rise in the vibriocidal antibody titre at 7^th^ day after the first and the second dose. Additional analyses were performed to compare all adverse events during the study period as well as geometric mean of reciprocal serum vibriocidal titres at baseline, day 7 and day 21 among vaccine and placebo recipients.

#### 2.4.2 Adverse Events

Adverse events of significance were diarrhea and vomiting that were solicited for 3 days after the first dose. Two weeks after the first dose, subjects returned to the clinical trial unit for the second dose and were asked to attend the clinic for follow-up daily for 3 days. Adverse events were evaluated during 21 days of the study period in both vaccine and placebo recipients. During each follow-up visit, study physicians conducted a structured interview regarding the subject’s over-all level of activity and bowel movements as well as occurrence of symptoms such as diarrhea, abdominal pain, loss of appetite, nausea, general ill feeling, fever (axillary temperature ≥98°F), headache or vomiting during the preceding 24 hrs. The stool consistency was ranked according to three grades: grade 1, firm or soft/mushy; grade 2, thick liquid; grade 3, watery. Three or more grade 2 stool or one or more grade 3 stool was defined as diarrhea. The attending physician took decision on the severity of diarrhea and graded them as mild with no dehydration, moderate with some dehydration and severe with marked dehydration.

### 2.5 Vibriocidal Assay

Five milliliters of venous blood was collected from the participants for vibriocidal assay prior to (-7 to -4 days) and 7 days and 21 days subsequent to vaccination. Blood group was determined with blood drawn on seven to 4 days prior to administration of the first dose of the vaccine during screening procedures. Vibriocidal assay was performed with the *V. cholerae* O1 Inaba (VA1.4) strain using sera collected during pre- and post-vaccine trial following the methods of Benenson and colleagues [5] which we described earlier [4]. Commercially prepared guinea pig serum was used as a complement in this study (Rockland Immunochemical Inc. Gilbertsville, PA, USA). A 4-fold or greater increase in titre between the −7 to −4 day of vaccination and days 7 and 21 sera samples was used to signify seroconversion.

#### 2.5.1 Anti-CT Assay

Enzyme-linked immunosorbent assay (ELISA) was used for the detection of antibody response against CT. Immunoglobulin G (IgG)-specific antibody response in the paired sera was determined against purified CT using micro titration plates (Nunc, Denmark). ELISA was made following the procedures of Nandy et al (1996) [6] with slight modification. We described the methods used for this study in our earlier communication [4].

#### 2.5.2 Excretion of Vaccine Strain

Stools for two consecutive days collected from the participants were examined and graded. Fecal excretion of vaccine strain was tested using conventional cultural and molecular methods. Stool specimens collected from the vaccine study participants were collected in sterile containers and transported to the laboratory and processed within 2 hrs of collection. Details of the method used in this study were described in our earlier communication [4], [7].

### 2.6 Sample Size

A one-sided 95% confidence interval was used to calculate the sample size in order to rule out clinically unacceptable high rates of diarrheal adverse event occurring during the 3 days after either dose as well as to establish adequate seroconversion to *V. cholerae* O1 Inaba among recipients. Assuming a 10% diarrheal rate among placebo and vaccine recipients alike, to exclude a vaccine-placebo difference in the rate of diarrhea of greater than 20% (upper boundary of the 1-tailed 95% confidence interval) with a power of 0.9, the minimum number of subjects required for each group was 39. For serum vibriocidal responses, assuming a background rate of 5% seroconversion among placebo recipients after one dose and a true vibriocidal response in the vaccine group of 60%, to exclude a vaccine-placebo difference of 30% (lower boundary of the 1-tailed 95% confidence interval) with a power of 0.9, the minimum of subjects required for each group was 40. To adjust for the number of persons expected to drop out of the study, at least 44 persons were therefore required in each group.

#### 2.6.1 Statistical Methods

Data analysis was performed using Stata version 11.0 (Stata Corp., Texas USA). Safety analysis was performed based on the number and percentage of subjects (with 95% CI) with at least one adverse event (solicited and/or unsolicited) after vaccination and during the 8 days and 21 days follow up period between the study groups. Analysis of immunogenicity was performed based on the number and percentage of adults (with 95% CI) exhibiting at least a fourfold rise in serum anti-O1 vibriocidal titre between the study groups. The geometric mean reciprocal titre and geometric mean reciprocal folds rise in titre over 7–8 days and 21 days were also compared between the vaccine and placebo groups. The proportion with ≥4 fold rise in titre (95% CI) was compared using Binomial Exact method.

#### 2.6.2 Investigational product

This vaccine was developed in three research laboratories of the Government of India and was not driven by the industry. The Department of Biotechnology of the Ministry of Science and Technology (DBT), Government of India was the lead agency. This vaccine was developed into a clinical product by Shantha Biotechnics Private Limited, Hyderbad, India for the present study and was supported by DBT. It is a single dose Lyophilized clinical product with 1 ml volume per vial after reconstitution. The Batch/Lot No. is CVM00611; its manufacturing date was Aug. 2011 and the stated expiry date was July 2012.

## Results

**Figure 1:**
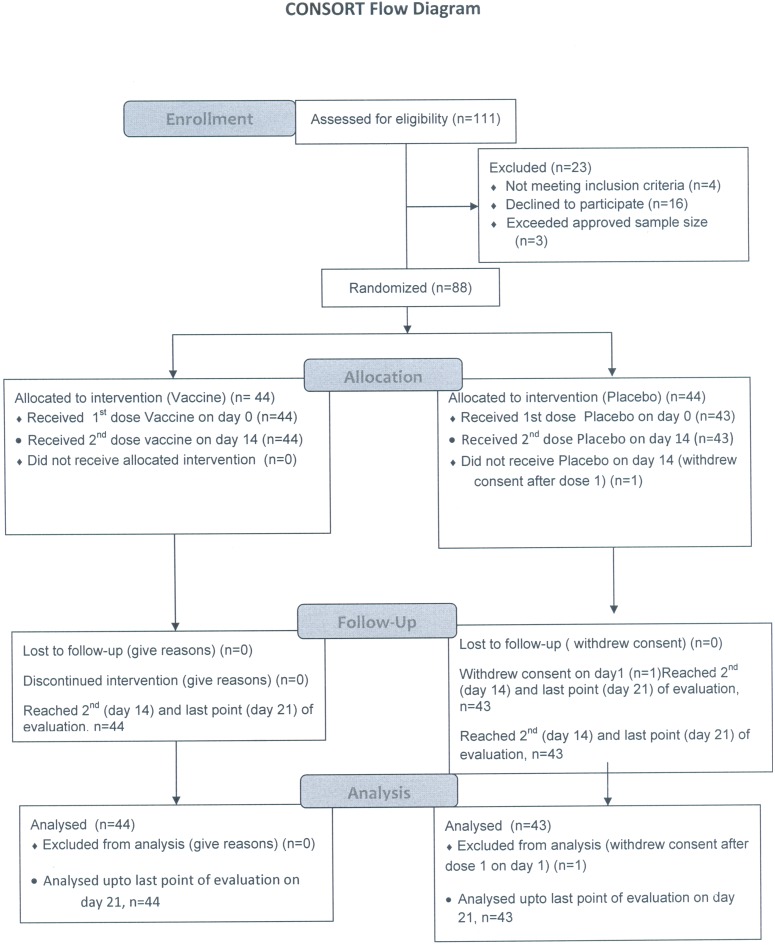
Consort Flow Diagram.

**Figure 2:**
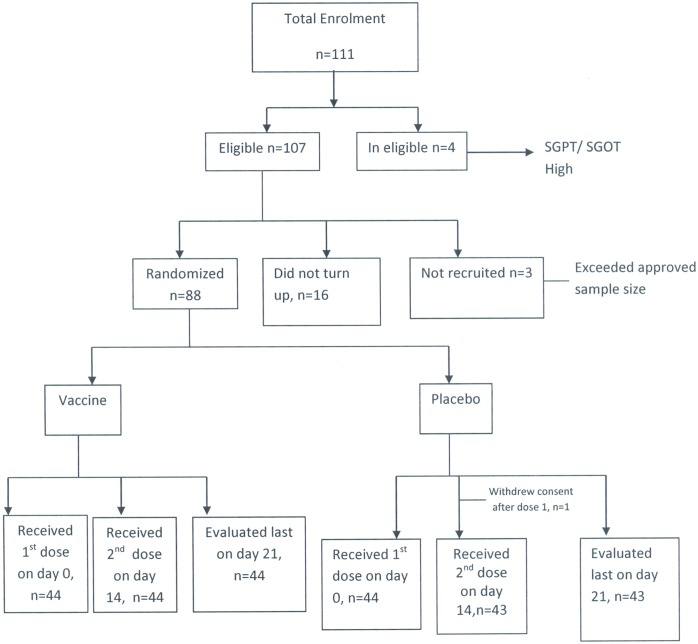
Flow chart for the recruitment of volunteers in the study.

Flowchart for enrolment and admission features of the volunteers is shown in **Figure 1**, **Figure 2** and **Table 2**. A total of 44 participants in the vaccine group and 44 in the placebo group were enrolled in this study. One participant in the placebo group withdrew consent soon after the first dose. The median age of vaccines was 31 years (range 19–51 years) compared to 32 years (range 19–57 years) among placebo recipients. Nearly 42% of the participants in the placebo arm belonged to blood group ‘O’ as compared to 30% in the vaccine arm.

**Table 2:** Admission features of the volunteers (n = 87).

Variables	Vaccine group (n = 44)	Placebo group (n = 43)
**Age**		
Median (quartiles)	32 (28, 38)	33 (25, 39)
18–28 years	13 (30%)	16 (37%)
29–39 years	20 (45%)	17 (40%)
40–50 years	8 (18%)	6 (14%)
>50 years	3 (7%)	4 (9%)
**Sex**		
Male	26 (59%)	26 (60%)
Female	18 (41%)	17 (40%)
**Weight (kg)**		
Median (quartiles)	55.6 (47, 70)	53.6 (48.15, 63.85)
**Hemoglobin (gm/dl)**		
Mean (SD)	12.74 (1.19)	13.2 (1.48)
Range	(10.3–15)	9.4–16.4
**Blood Group**		
Group O	13 (30%)	18 (42%)
Group A	14 (32%)	10 (23%)
Group B	15 (34%)	11 (26%)
Group AB	2 (4%)	4 (9%)

### 3.1 Adverse events

Adverse events were rare (**Table 3**). None had nausea, vomiting or diarrhea. No adverse events occurred more frequently in vaccine group than in placebo group. No serious adverse event (SAE) occurred in any subject.

**Table 3:** Comparison of solicited adverse events following the first and second doses of vaccine (n = 44) and placebo group (n = 43).

	Within 3 days after the 1^st^ dose		Within 3 days after the 2^nd^ dose	
	Vaccine	Placebo	Vaccine	Placebo
Nausea	0	0	0	0
Vomiting	0	0	0	0
Diarrhoea	0	0	0	0
Rash	0	0	0	0
Abdominal pain	1	0	0	1
Loss of appetite	1	0	0	0
General ill feeling	1	0	0	1
Fever	0	1	1	1
Headache	0	0	1	1
Cough	0	1	0	0
Backache	1	0	0	0

### 3.2 Immune Response: Vibriocidal Antibody Titre

Serum vibriocidal antibody titre to *V. cholerae* O1 at baseline, day 7 and day 21 are shown in **Table 4**. The geometric mean reciprocal titre on day 7 and day 21 in the placebo group were similar to baseline titre. In the vaccine group the mean geometric reciprocal titre on day 7 and day 21 were similar and both were more than 7 folds higher than that at base line. The proportion of subjects with ≥4 folds rise in titres in the two groups are shown in **Table 5**. The proportion of participants who seroconverted (i.e. had 4-folds or higher rise in reciprocal titre) in the vaccine group were 65.9% (95% CI: 50.1%–79.5%) at both 7 days (i.e. after 1^st^ dose) and 21 days (i.e. after 2^nd^ dose). None of the placebo recipients seroconverted. In the subgroup of participants belonging to blood group O (**Table 6**) the proportion of subjects with ≥4 folds rise in vibriocidal antibody titre were 53.8% (95%, CI: 25.1%–80.8%) and 69.2% (95%, CI: 38.6%–90.9%) on day 7 and day 21, respectively.

**Table 4:** Serum vibriocidal antibody titres to Vibrio Cholerae 01 at baseline, day 7 and day21.

GMT^a^	Vaccine, n = 44 (95%CI)	Placebo, n = 43 (95%CI)	P
**Baseline**	205.9 (117.1 to 361.9)	147.6 (87.1 to 250.1)	0.39
**Day7**	1546.4 (1080.0 to 2214.0)	152.5 (90.0 to 258.3)	<0.001
**Day21**	1498.4 (1094.1 to 2052.0)	154.9 (90.9 to 263.9)	<0.001
**GMF** b **rise**			
**Baseline to day**	**7**7.5 (4.56 to 12.38)	1.0 (0.99 to 1.08)	<0.001
**Baseline to day 21**	7.3 (4.34 to 12.21)	1.1 (1.05 to 0.99)	<0.001

a GMT: geometric mean reciprocal titre.

b GMF: geometric mean reciprocal titre: folds rise over baseline.

**Table 5:** Rise in vibriocidal antibody titre between baseline and day 7 and baseline and day 21 of vaccine (n = 44) and placebo (n = 43) recipients.

Number with	Baseline to day7			Baseline to day21		
	Vaccine (n = 44)	Placebo (n = 43)	P-value	Vaccine (n = 44)	Placebo (n = 43)	P-value
**No rise**	6 (14%)	41 (95%)		6 (14%)	40 (93%)	
**2-fold rise**	9 (20%)	2 (5%)		9 (20%)	3 (7%)	
**4-fold rise**	7 (16%)	0		9 (20%)	0	
**8-fold rise**	9 (20%)	0		8 (18%)	0	
**≥16-fold rise**	13 (30%)	0		12 (28%)	0	
**Number of subjects who seroconverted (%)** a	29 (65.9%)	0	<0.001	29 (65.9%)	0	<0.001
**95% confidence interval** b	**(**50.1–79.5%)	(0–8.2%)		(50.1–79.5%)	(0–8.2%)	

a Number of subjects with ≥4-fold rise in titres from baseline to day 7 and day 21.

b Binomial, exact.

**Table 6:** Rise in vibriocidal antibody titre between baseline and day7 and baseline and day21 of vaccine (n = 13) and placebo (n = 18) recipients among blood group O.

Number with	Baseline to day7			Baseline to day21		
	Vaccine (n = 13)	Placebo (n = 18)	P-value	Vaccine	Placebo	P-value
**No rise**	2 (15%)	16 (89%)		2 (15%)	15 (83%)	
**2-fold rise**	4 (31%)	2 (11%)		2 (15%)	3 (17%)	
**4-fold rise**	0	0		3 (23%)	0	
**8-fold rise**	4 (31%)	0		2 (16%)	0	
**≥16-fold rise**	3(23%)	0		4 (31%)	0	
**Number of subjects who seroconverted (%)** a	7 (53.8%)	0	0.0059	(69.2%)	0	0.002
**95% confidence interval** b	**(**25.1–80.8%)	(0–18.5%)		(38.6–90.9%)	(0–18.5%)	

a Number of subjects with ≥4-fold rise in titres from baseline to day 7 and day 21.

b Binomial, exact.

#### 3.2.1 Anti-CT antibody titre

No significant rise in antibody titre from baseline values was noted against CT (anti-CT antibodies) in the vaccine group at 7 days and 21 days of vaccination (**Table 7**). Similarly, no significant change in the geometric mean reciprocal titre of anti-CT antibody was noted at days 7 and 21.

**Table 7:** Rise in anti-CT antibodies between baseline and day7 and baseline and day21 of vaccine (n = 44) and placebo (n = 43) recipients.

Number with	Baseline to day7			Baseline to day21		
	Vaccine	Placebo	P-Value	Vaccine	Placebo	P-Value
**No rise**	41 (93%)	43(100%)		38(85%)	42(98%)	
**≥2-fold rise**	3 (7%)	0	0.09	6(15%)	1(2%)	0.07
++Relative risk (95%CL)	0.93 (0.00 to 2.44)			0.88 (0.00 to 1.35)		
**1 tailed P-value	0.125			0.059		
*GMT	Vaccine group (n = 44)			Placebo group (n = 43)		
Baseline	1498.4			1387.4		
Day 7	1321.0			1005.1		
Day 21	1475.0			989.0		

*GMT: geometric mean reciprocal titre.

++ CL: Exact confidence limits (Mehta, Patel and Gray 1985).

**Fisher exact.

#### 3.2.2 Fecal excretion of the vaccine strain

One subject in the vaccine group excreted the vaccine strain on day 1 (i.e. 24 hours after vaccination). The subject did not show any adverse event.

## Discussion

In this study we evaluated a live oral cholera vaccine VA 1.4, identical to VA 1.3 studied earlier [4] except for the ampicillin resistance marker which has been deleted. The vibriocidal antibody response to VA 1.4, a surrogate marker of protection, was at least as good as its precursor. In a study in the same population a 2-dose killed oral cholera vaccine [8] which is now a licensed vaccine showed a seroconversion rate of 53% (lower 95% CI being 36%), which is substantially lower than this single dose live oral vaccine (66%, lower 95% CI 50%). As has also been reported for the two dose killed oral cholera vaccine evaluated in the same population [8] and for another live oral vaccine Peru 15 in a similarly endemic population [9] the response is inversely related to the baseline vibriocidal titre (p = 0.001). Adverse events were rare and mild and none had diarrhea or vomiting after taking the vaccine.

We may note, unlike its precursor (VA 1.3) the VA 1.4 does not induce good anti-CT antibody response. It could be because unlike VA1.3, VA 1.4 harbors a single copy of *ctx*B. However, it may be pointed out that unlike vibriocidal antibody titre anti-CT antibody titre is not regarded a marker of protection against cholera. Similar indifferent anti-CT antibody response to another live oral candidate cholera vaccine has been reported [9], [10].

Serum vibriocidal antibody titre as a marker of protection was observed over four decades of epidemiological studies and vaccine efficacy trials [11]–[15] and a general consensus emerged that a high vibriocidal antibody response is a good marker of protection. Challenge studies in human volunteers showed that the degree of stimulation of serum vibriocidal antibody following ingestion of a live oral cholera vaccine is a good correlate of the antibacterial immunity in the intestine. However, vibriocidal antibody titre is considered an incomplete and surrogate marker of the not so well understood immune responses that are more directly related to protection [16]–[18]. A better understanding of the specific mechanisms of immunity to cholera is yet to be established. Studies in animals have shown that cholera toxin, toxoid, or B subunit of cholera toxin induces high levels of serum antitoxin and provides protection against cholera, albeit a short-lived one because, serum anti-toxin declines rapidly [19]–[23]. However, two separate field trials of parenteral toxoid vaccines in Bangladesh and the Philippines provided little evidence of protection [17], [24].

One subject in the vaccine group excreted the vaccine on day1 (i.e. 24 hours after the first dose of the vaccine). The subject did not show any adverse event. A more general concern may be that an attenuated cholera vaccine strain could acquire *ctxA* gene from a wild type *V. cholerae* in the intestine or in the environment. If such a transfer occurs, adverse consequence if any should be minimal. Firstly, there should already be a large number of toxigenic *V. cholerae* present in the intestine representing a co-infection or a toxigenic strain present in the environment to serve as a source of *ctxA*. Adding one more toxigenic strain of *V. cholerae* in the environment or in a cholera patient excreting up to 10 L cholera stool containing 10^7^ −10^8^ toxigenic *V. cholerae* per ml would be insignificant. Another live oral cholera vaccine CVD-103 has been tested in a variety of settings including a large field trial in Indonesia where cholera is endemic and has had an excellent safety record. Like all *microbes V. cholerae* can evolve and acquire new attributes and a rare event like re-acquiring *ctxA* by an attenuated cholera vaccine strain is not significant compared to the normal evolutionary changes in a wild type pathogen.

Regarding the shelf life of this live oral lyophilized cholera vaccine it may be noted that it was administered in the 11^th^ and 12^th^ month after it was manufactured. This situation has provided us with a serendipitous finding that it has at least 12 months of effective shelf life. This aspect needs to be explored further.

Only known host for cholera are humans. It largely spreads by fecal contamination of food and water. Recently in many countries, particularly in Africa protracted cholera outbreaks occurred. Of particular concern is the epidemic in Zimbabwe which lasted nearly a year. While overall cholera case fatality rate is <5%, in some situations it may reach 50% among vulnerable groups. One other recent event is of concern. In recent years a new variant of *V. cholerea* El Tor emerged [25]. This variant produces cholera toxin of the type produced by the classical cholera biotype. This variant has already replaced original El Tor strain in several parts of Asia and Africa and, appears to be associated with more severe disease. All these events therefore indicate the need for an effective single dose oral vaccine for cholera to combat cholera outbreaks and it is hoped that VA1.4 may meet this requirement.

## Supporting Information

Checklist S1 **CONSORT Checklist.**
(DOCX)

Protocol S1 **Trial Protocol.**
(PDF)
