# Supplementary material for: Safety and Immunogenicity of a Live Oral Recombinant Cholera Vaccine VA1.4: A Randomized, Placebo Controlled Trial in Healthy Adults in a Cholera Endemic Area in Kolkata, India
Source: PLoS One. 2014 Jul 1;9(7):e99381. doi: 10.1371/journal.pone.0099381 (PMC4077646; doi:10.1371/journal.pone.0099381)
Supplement: Protocol S1 — Trial Protocol. (PDF) [file pone.0099381.s002.pdf]

Trial of Candidate Live Oral Cholera Vaccine VA 1.4: A Phase I-II Study to Evaluate the Safety and Immunogenicity in Healthy Adults in Kolkata, India

Protocol No. 1, Version No. 1.8.3
Date: 27.04.2012

The information in this document should be treated as confidential, not to be divulged to unauthorized person (s) in any form, including publication(s) and presentation(s), without the expressed written consent of Department of Biotechnology (DBT), India.

1. GENERAL INFORMATION

1.1. Trial of Candidate Live Oral Cholera Vaccine VA 1.4: A Phase I-II Study to Evaluate the Safety and Immunogenicity in Healthy Adults in Kolkata, India

Protocol No. 1, Version No. 1.8.3
Date: 27.04.2012
V/1.8.2/23.3.2012

Confidentiality Clause: Confidential information is provided to you as a concerned person. By accepting this document you agree that the information contained herein will not be disclosed to others, without written authorization from the Society for Applied Studies and The National Institute of Cholera and Enteric Diseases, Kolkata.

1.2. Sponsor

- Department of Biotechnology (DBT), Ministry of Science and Technology, Government of India, CGO Complex, Lodhi Road, New Delhi, India

1.3. Person authorized to sign the protocol

- Dr. Dilip Mahalanabis, Director, Society for Applied Studies, Kolkata

1.4. Onsite Clinical Monitor

- Dr. Santi Ranjan Bagchi (Rtd. Professor & Principal, ID & BG Hospital, Beliaghata, Kolkata-700 010; Mobile: 9432214599

1.5. Investigators

1.5.1. Principal Investigators

Dr. Dilip Mahalanabis, Society for Applied Studies, CF-198, Salt Lake, Sector 1, Kolkata-700 064; Phone: +91-33-2358 8850 (O), +91-33-2337 2562 (H), E-mail: sascf198@yahoo.com

1.5.2. Collaborating Investigators

- Dr. T. Ramamurthy, NICED, Kolkata
- Dr Byomkesh Manna, NICED, Kolkata
- Dr. Dipika Sur, NICED, Kolkata
- Dr. Suman Kanungo, NICED, Kolkata
- Dr. Mihir Kumar Bhattacharya, NICED, Kolkata
- Dr. Bandana Sen, SAS, Kolkata

1.5.3. Project Advisors

- Dr. Bindu Dey, Adviser, Department of Biotechnology, New Delhi
- Dr. G. Balakrish Nair, Executive Director, THSTI, New Delhi
- Dr. Amit Ghosh, NICED, Kolkata

1.5.4. Laboratory (Microbiology & Clinical)

- Laboratory of the National Institute of Cholera and Enteric Diseases, P-35, C.I.T Road, Scheme-XM, Beliaghata, Kolkata-700 010; Phone: +91-33-2363-3373

1.5.5. Study Site

- The study will be conducted at the Collaborative Clinical Research Unit of the Society for Applied Studies and the National Institute of Cholera and Enteric Diseases, located in the Infectious Diseases Hospital Premises, 57A, S.C.Banerjee Road, Kolkata-700010, Kolkata, India.

TABLE OF CONTENT

	Page No.
1. STUDY SYNOPSIS	4
2. BACKGROUND INFORMATION	6
3. RATIONALE AND OBJECTIVE	8
4. TRIAL DESIGN	8
5. SELECTION AND WITHDRAWAL SUBJECTS	14
6. TREATMENT OF SUBJECT	15
7. ASSESSMENT OF EFFICACY	16
8. SAFETY	16
9. STATISTICS	18
10. ACCESS TO SOURCE DATA/DOCUMENTS	19
11. QUALITY CONTROL AND QUALITY ASSURANCE	20
12. ETHICS	20
13. DATA HANDLING AND RECORD KEEPING	20
14. FINANCING AND INSURANCE	21
15. PUBLICATION	21
16. REFERENCE LIST	22
17. ANNEXURES	23

STUDY SYNOPSIS

Title: Trial of Candidate Live Oral Cholera Vaccine VA 1.4: A Phase I-II Study to Evaluate the Safety and Immunogenicity in Healthy Adults in Kolkata, India

Study Type: Randomized Controlled Trial, Phase I-II.

Study Objective:

a) To evaluate the safety and immunogenicity of live oral **VA1.4** cholera vaccine (identical to VA1.3 except for absence of Ampicillin marker) in adult volunteers in Kolkata, India, aged 18 years to 60 years.

Location: The study will be conducted at the Clinical Research Unit of the Society for Applied Studies (SAS) located in the Infectious Diseases & Beliaghata General Hospital Premises, 57A, S.C Banerjee Road, Beliaghata, Kolkata - 700010, India.

Study Design: Randomized double blind placebo controlled trial in adults.

Study Subjects: Healthy adult men and non-pregnant women aged 18 to 60 years will be recruited for the study.

Sample Size:

a) Healthy adult men and non-pregnant women aged 18 to 60 years, 88 in number (44 in the Vaccine arm and 44 in the placebo arm).

Study Vaccine: Live Oral Cholera Vaccine **VA 1.4** (Lyophilized single dose vials, Identical to VA1.3 except for Ampicillin marker) (using lyophilized single dose pack manufactured as cGMP lot)

Evaluation Criteria:

Safety: Proportion with adverse diarrheal events, i.e. the adverse events in the vaccine group will be compared with that occurring in the placebo group.

Immunogenicity: Seroconversion rate (i.e. ≥4 fold rise in vibriocidal antibody titre) at 7 days to 9 days after the first dose of vaccine and again on 7th day after the second dose (on day 14).

Study Period: Vaccine trial: 6 months

	Size	Day 0	Day 14
ADULTS	44	Vaccine	Vaccine
	44	Placebo	Placebo

FLOWCHART (Schedule of visits)

88 subjects in the study population (88 individuals, healthy, non-pregnant adults of aged 18 years to 60 years):

Study Procedure	Day -7 to -4	Day 0	Day 1 &2	Day 3	Day 7	Day 14	Day 15-16	Day 17	Day 21
Informed Consent	X								
History and Physical Exam	X								
Screening	X								
Clinical Evaluation	X		X		X	X	X		X
Randomization	X								
Blood Draw - Lab Tests#	X								X
Blood Draw - Vibriocidal Assay	X				X				X
Administration of Study Agents- **Oral Cholera Vaccine VA1.4** or **Placebo**		x				X			
Stool/Rectal Swab - Shedding			X						
Solicited Symptoms (Reactogenicity symptoms)	X		X	X	X	X	X	X	X
Adverse Event Monitoring	X		X		X	X	X		X

Routine Blood Test [Hb, TLC, DLC, Platelets]
KFT [Urea, Creatinine, Electrolyte (Na^+, K^+), Uric Acid]
LFT [SGOT, SGPT, ALP, Total Proteins & Albumin, Total Bilirubin]

2. BACKGROUND INFORMATION

2.1. Study Vaccine

Live Oral Cholera Vaccine **VA 1.4**
(Identical to VA1.3 except for Ampicillin marker)

2.2. Background

Cholera is endemic in many countries, particularly in South and South East Asia. An effective Cholera Vaccine has substantial public health relevance for these countries. With increasing global travel such a vaccine may have use for travelers to cholera affected regions. A single dose live oral vaccine may also help control of cholera outbreaks There is one other licensed live oral cholera vaccine i.e. **CVD 103 HgR** (*following unsatisfactory results of a field trial of this vaccine in Indonesia*). This is largely used for travelers and has been marketed. Recently its production has been discontinued. A killed oral cholera vaccine with added B-subunit of cholera toxin has also been licensed and marketed mainly for travelers. This vaccine has so far not been exploited for large-scale public health use. A simpler version of a killed oral cholera vaccine without B-subunit has however been tested and implemented in Vietnam with reported success in reducing severe disease from cholera (Thungapathra M et.al. 1999). A version of this vaccine is undergoing field testing in India. This is a two dose vaccine.

A new oral candidate vaccine has been constructed from a non-toxigenic strain of *Vibrio cholerae* El Tor, Inaba, which is not only devoid of the cholera toxin (CT) virulence cassette but also is completely non-reactogenic in rabbit ileal loop assay and has been described (*see Appendix-I*). The strain, however, has *tox*R and *tcp*A genes. Through a series of manipulations, the *ctx*B gene of *V. cholerae*, responsible for the production of the 'B' subunit of the cholera toxin (CTB) was introduced into the cryptic haemolysin locus of the strain. The resulting strain, named vaccine attempt 1.3 (VA1.3), was found to be able to produce copious amounts of CTB. In the RITARD model this strain was found to be non-reactogenic and provided full protection against the challenge doses of both *V. cholerae* O1, classical and El Tor. In the immunized rabbit it evoked significant levels of anti-bacterial and anti-toxin immunity. In a randomized placebo controlled trial, we evaluated this vaccine in 320 human volunteers from Kolkata, India, over a period from 1999 to 2005 for safety and immune response. Kolkata is known to be a highly endemic zone for cholera. Recently, we also studied a group of volunteers from a less endemic zone in Vellore in South India for safety and immune response, using a similar protocol.

In a large human volunteer study with Live Oral Cholera Vaccine VA1.3 we have shown that seroconversion rate with this novel vaccine is excellent and the adverse effects are negligible. Further, the vaccine strain is not excreted in the stool. The results of this study are summarized in (ref. 8). This vaccine strain however has an Ampicillin marker and it was introduced for easy detection in the environment. Following advice from some experts the developers of the vaccine have now deleted the marker and the strain is named VA1.4. This construct is identical to VA1.3 except for the absence of the Ampicillin marker. **VA1.4** is now being developed into a clinical product following cGMP by Shantha Biotechnics Pvt. Ltd., Hyderabad. **The present proposal is a Phase I to II study to evaluate the safety and immunogenicity of VA1.4 (an identical construct to VA1.3).**

Regulatory Requirements

1. Acute toxicity safety, reactogenicity and immunogenicity of the live oral recombinant vaccine strain VA 1.3 was carried out in animals (Rabbit and infant mouse) at PGIMER, Chandigarh. Animal experiments for determining reactogenicity and immunogenicity of the vaccine strain VA 1.3 in the rabbit model RITARD was carried out at National Institute of Cholera and Enteric Diseases (NICED), Calcutta. VA1.3 was found to be safe and immunogenic in animal models. **Annexure I**

2. In a letter dated 17 June 1997 (No. 65/15/Misc/92-ECD-II), DCGI & Technical Committee of the ICMR approved the phase I-II human volunteer study with VA1.3 human volunteer studies as per proposal sent to them. **Annexure II**

3. VA1.3 was also found to be safe and immunogenic in human. The data was published in *Vaccine* and is placed at **Annexure III**.

4. The VA1.4, the Investigational New Product, IP which is only a slightly modified version of VA1.3 in form of Amp. Marker removal. It has also undergone toxicological studies as per the schedule Y of the Drugs & Cosmetics Act and following Bio-safety norms of the genetically modified organisms. The toxicological studies have been done at an accredited laboratory i.e. Cadila Pharmaceuticals Ltd as per the approval of the batch-data specifications of the Review Committee on Genetic Manipulation (RCGM). **Annexure IV**

5. The data has been seen by the RCGM and approval accorded to conduct the Phase I/II clinical trials. Letter is enclosed at **Annexure V**.

6. The batch for the trials as prepared by the Shantha Biotechnics Pvt. Ltd., Hyderabad has been cleared by CDL. The letter is placed at **Annexure VI**.

7. The study has been cleared by the Scientific Advisory Committee (SAC), Institutional Biosafety Committee and Institutional Review Committee (IEC) of NICED and the Ethics Review Committee of Society for Applied Studies. **Annexure VII**

2.3 Approval for human volunteer study by the Drugs Controller General (India)

Department of Biotechnology, Ministry of Science & Technology has contracted Shantha Biotechnics Pvt. Ltd. to produce the live oral cholera vaccine VA1.4 as a clinical material using cGMP and in conformity with relevant regulatory requirements. The product will be in the form of a single dose lyophilized vial containing the vaccine. This clinical product is for use in Phase I-II and Phase III field trials. Identical placebo vials will also be manufactured and provided by Shantha Biotechnics Pvt. Ltd. The protocol has been submitted to DCGI for approval for human volunteer study using VA1.4 cholera prepared by Shantha Biotech as cGMP lot and got approval.

2.4. Vaccine administration

The Vaccine (single dose lyophilized clinical product) will be administered orally as a reconstituted single dose, with a dose of sodibicarb solution buffer prior to administration; this is to neutralize the stomach acid. A single dose oral vaccine for protection from a disease is an optimum mode of administration. The control group will receive an identical looking reconstituted lyophilized placebo vial containing excepients only. The subject will drink a buffer solution (bicarbonates 2.5g and ascorbic acid 1.65g in 100 ml) before administration of vaccine/placebo. The freeze dried vaccine or placebo vials will be reconstituted in 1ml of diluents provided by the manufacturer and given directly into month to drink 5 minutes after drinking the buffer solution.

2.5. Statement

We hereby state that the trial will be conducted in compliance with the protocol, GCP, and applicable regulatory requirements.

2.6. Study Population

Healthy adult men and non-pregnant women (aged 18-60 yr) from Kolkata will be enrolled for the study.

3. RATIONALE AND OBJECTIVE

3.1. Rationale

We conducted a large volunteer study of Live Oral Cholera Vaccine VA1.3 and found it largely free from adverse effects and it produces excellent immune response (Randomized placebo controlled human volunteer trial of a live oral cholera vaccine VA1.3 for safety and immune response. Mahalanabis D, Ramamurthy T, Nair GB, Ghosh A, Shaikh S, Sen B, Thungapathra M, Ghosh RK, Pazhani GP, Nandy RK, Jana S, Bhattacharya SK Vaccine. 2009 Jul 30;27(35):4850-6. Epub 2009 Jun 10). VA1.3 has an Ampicillin resistance marker for easy detection. Following on expert advice this marker was deleted to form an identical vaccine without the marker now named VA1.4. This Phase I-II study is therefore important to confirm that adverse events are few or none and has similar immune response (Mahalabis *et al.* 2009). The results will help expedite and pave the way for field study of the clinical product (vaccine).

3.2. Objectives

(i) To evaluate the safety and immunogenicity of the live oral cholera vaccine **VA1.4 (IP)** in adult volunteers of either sex in Kolkata (following both one and two doses).

4. TRIAL DESIGN

4.1. End Points

I. In this trial in adults we will compare the live oral cholera vaccine and placebo recipients for: I occurrence of adverse events to show safety, e.g. diarrhea, vomiting, fever, abdominal pain or cramps, headache, loss of appetite, general ill feeling, rash; and

II. Serum vibriocidal antibody response (≥4 fold geometric mean folds rise) to serogroup 01, to show immunogenicity.

4.2. Trial: After examination of the safety data in adults (i.e. on first consecutive 40 enrolled) and on receipt of DCGI clearance based on safety data, the trial protocol in children will be developed and submitted to DCGI for clearance. The trial will be randomized double blind and placebo controlled. Randomization ratio will be 1:1 of vaccine and placebo.

Healthy adult men and women (aged 18 to 60 years) will be randomized to receive a single dose of either a live oral cholera vaccine VA1.4 or a placebo dose with the diluents used for constituting the vaccine dose as par manufacturer's instruction. Each agent (vaccine or placebo) will be administrated to volunteers on day 0 and observed for a day at the research unit and on 2 more consecutive days with clinic visits with extended stay (9 am to 4 pm). Blood samples (10 ml) will be obtained on day -7 to -4 days (prior to administering the agent) for baseline vibrocidal and laboratory assessment. Primary end points are the occurrence of adverse events and serum vibriocidal response. Solicited adverse events will be noted down on day 0-1-2 and on day 7 or 8. On Day 7, Blood samples (3-5 ml) will be obtained for Vibrocidal assessment. On day 14, a second dose of vaccine or placebo would be administered following the same procedure as on day 0. The code for the volunteers on day 14 will remain same as that on day 0. Solicited symptoms and/or adverse events will be recorded on day 15, 16 and 17.

Follow ups of the subjects for the reactogenicity will be done at the clinic or through home visit by the physicians and the health workers recruited for the project. The subject will drink a buffer solution (bicarbonates 2.5g and ascorbic acid and 1.65g) in 100 ml, 5 minutes prior to drinking the vaccine dose. On day 21 third blood sample (10 ml) will be obtained for vibrocidal and safety assessment.

4.2.1 Flow Chart

**Study Procedure**	Day -7 to -4	Day 0	Day 1 &2	Day 3	Day 7	Day 14	Day 15-16	Day 17	Day 21
Informed Consent	X								
History and Physical Exam	X								
Screening	X								
Clinical Evaluation	X		X		X	X	X		X
Randomization	X								
Blood Draw - Lab Tests[#]	X								X
Blood Draw - Vibriocidal Assay	X				X				X
Administration of Study Agents- **Oral Cholera Vaccine VA1.4** or **Placebo**		x				X			
Stool/Rectal Swab - Shedding			X						
Solicited Symptoms (Reactogenicity symptoms)	X		X	X	X	X	X	X	X
Adverse Event Monitoring	X		X		X	X	X		X

Routine Blood Test [Hb, TLC, DLC, Platelets]
KFT [Urea, Creatinine, Electrolyte (Na^+, K^+), Uric Acid]
LFT [SGOT, SGPT, ALP, Total Proteins & Albumin, Total Bilirubin]

4.2.2 STUDY PROCEDURES

Schedule and description of observations and visits
For 88 subjects in study population (Adults):

a. **Day -7 to -4:** Informed consent is to be obtained from the subject. Next, all consenting subjects will be assigned a unique Study ID number, in consecutive sequence. This means that all consenting subjects will receive an ID number, regardless of whether they are medically eligible or ineligible. Next, screening for inclusion and exclusion criteria, history and physical examination are completed by the study physicians. Then approximately 10 ml of blood will be obtained for baseline (pre-immunization) immunologic and laboratory evaluation.
b. **Day 0**: Once the subject is included based on the laboratory reports and clinical assessment, a **study serial number** will be assigned. Subjects will be randomized following the randomization list. Subjects will be logged into the randomization list in a sequential manner. The study agent will be administered according to the assigned randomization number. The subjects will be asked to stay in the clinic (day care unit) for 6 hours for solicited and adverse event monitoring. CRF 1 Day 0 will be completed.

c. **<u>Day 1-2:</u>** Subjects will be followed-up for interval solicited adverse event monitoring (either they return to the center or are visited at home). Stool/ Rectal Swab will be collected for shedding. CRF 1 Day 1 and Day 2 will be completed.
d. **<u>Day 3:</u>** Subjects will be followed-up for any solicited symptoms. CRF 1 Day 3, will be completed (either clinic or home visits).
e. **<u>Day 7:</u>** Subjects will be evaluated clinically. Then approximately 3-5 ml of blood will be obtained for immunologic response. CRF 1 Day 7 will be completed.
f. **<u>Day 14</u>**: Subjects will return to the study center for the second dose. The second dose of study agent will be given according to the assigned randomization number. The code for the volunteers on day 14 will remain same as that on day 0. The subject will be asked to wait in the clinic for 4 hours for adverse event monitoring. CRF 1 Day 14 will be completed.
g. **<u>Day 15-16</u>**: Subjects will be followed-up for interval solicited adverse event monitoring (either they return to the center or are visited at home). CRF 1 Day 15 and Day 16 will be completed.
h. **<u>Days 17</u>**: Subjects will be followed-up for any solicited symptoms. CRF 1 Day 17 will be completed.
i. **<u>Day 21</u>**: Subjects will be evaluated clinically. Then approximately 10 ml of blood will be obtained for immunologic response and safety assessment. CRF 1 Day 21 will be completed. The Study Summary will be completed.

Assessment after vaccination

4.2.3 Follow-up for Adverse Events

Following each dose, subjects will be observed in the clinic (vaccinating area) for 4 hours to assess for any immediate reactions.

After each dose of the study agent, subjects will be followed up on an out-patient basis (either asked to return to the center or followed up at home) for three days. The subject will provide a 24-hour recall history of symptoms and temperature will be taken. Study staff will complete the appropriate CRF pages. Any unsolicited adverse events will be documented on CRF Appendix for Adverse Events.

In addition to the three days of follow up following each dose, interval clinical evaluation will be performed on Day 7, and 21 to assess for any adverse events that may have occurred. Any adverse events noted will be recorded on CRF Appendix for Adverse Events. Any medications taken, non- solicited Adverse Events and Serious Adverse Events reported during the study period will be recorded on the appropriate Appendix pages of the CRF.

Follow-up for Serious Adverse Events

Any serious adverse event which occurs during a subject's participation in the study will be reported using the Serious Adverse Events Form in the Appendix pages of the CRF. A serious adverse event (experience) is any untoward medical occurrence that at any dose:

- Results in death,
- Is life-threatening. The term "life-threatening" in the definition of "serious" refers to an event in which the subject was at risk of death at the time of the event; it does not refer to an event, which hypothetically might have caused death, if it were more severe.
- Requires in subject hospitalization or prolongation of existing hospitalization,
- Results in persistent or significant disability/incapacity,
- Any other important medical event that may not be immediately life-threatening or result in death or hospitalization but may jeopardize the subject or may require medical or surgical intervention to prevent one of the other outcomes listed in the definition above. Prudent medical judgment must be exercised to decide whether reporting is appropriate. An example includes treatment for allergic bronchospasm that does not result in hospitalization but required intensive medical intervention in the emergency room.

Medical and scientific judgment should be exercised in deciding whether expedited reporting is appropriate in other situations, such as important medical events that may not be immediately life-threatening or result in death or hospitalization but may jeopardize the subject or may require

intervention to prevent one of the other outcomes listed in the definition above. These should also usually be considered serious.

4.2.4 Blood Draws

For 88 subjects in study group:

Venipuncture to draw approximately 10 ml blood prior to dose 1, 3-5 ml at 7 days after dose 1 and 10 ml at 7 days after dose 2 will be performed for the 88 subjects in the study group. Each specimen will be labeled with the following information: date of blood draw, initials, and study ID number.

At the time of the first bleed, -7 to day 0, the CRF containing the prescreening information will be filled up to indicate success of blood collection. Then CRF Day 0 will be completed to indicate the success of the dosing. At the time of second bleed CRF 1 Day 7 will be completed and at the time of the third bleed CRF 1 Day 21 will be completed, in a similar manner. During all the blood draws, numbered laboratory stickers will be affixed to the corresponding CRF and specimen.

4.2.5 Stool Samples/Rectal Swab

For the 88 subjects for assessment of other immunologic assays only:

Stool samples/Rectal Swab will be collected after dose 1, on day 1 and 2 for all the subjects for assessment of shedding of the vaccine strain. Each specimen will be labeled with the following information: date of stool collection, subject initials and study ID number.

CRF Day 1 and 2 will be completed to indicate whether each stool sample was provided. For all stool samples numbered laboratory stickers will be affixed to the corresponding CRF and specimen.

4.2.6 Sample Storage

After study testing, and in accordance with subject instruction obtained during informed consent, remaining samples may be destroyed or stored for future use.

4.2.7 Laboratory procedures

Serum vibriocidal antibody assay

The vibriocidal antibody assay is a bactericidal assay requiring the presence of complement-fixing antibody bound specifically to vibrios; this serum antibody response increases after clinical cholera or after vaccination. The serum samples from the volunteer prior to immunization and 7 days after each dose will be tested using vibriocidal antibody assay. An increase of titer by 4-fold or greater between baseline and post-immunization sera will be considered a significant antibody response. The vibriocidal assay using the microtiter technique will be performed in the NICED. As a secondary outcome measure vibriocidal antibody titre will be measured in the sample taken on day 21 to compare with baseline titre and with titre on day 7.

Vibriocidal assay

About five ml of venous blood will be collected from the volunteers for vibriocidal assay prior to (0-day) and 7 days and 21 days subsequent to vaccination. Blood group will be determined with blood drawn on 0-day. This will be done as it is known that blood group O+ve are prone to get cholera more often, this could be a potential confounder. Vibriocidal assay will be performed with the *V. cholerae* O1 Inaba (VA.1.3) strain using sera collected during pre and post vaccine trial following the published methods [6]. Commercially prepared guinea pig serum was used as a complement in this study (Sigma, St. Louis, USA). The sera (100 μl) will be added to 100 μl of PBS in the first well to give 2 fold dilution and the subsequent dilutions will be made reciprocally up to 4,800. A fourfold or greater increase in titer between the 0-day and 15th day sera samples will be used to signify sero-conversion. Reference rabbit antiserum against O1 Inaba will be included in each assay as a control. In addition, in every batch of the assay, serum obtained from a healthy volunteer who never had cholera and a high titer antiserum

obtained from one of the volunteers in this study will be included as negative and positive controls, respectively.

Anti-CT assay

Enzyme-linked immunosorbent assay (ELISA) will be used for the detection of response against CT antibody. Immunoglobulin G (IgG)-specific antibody response in the paired sera will be determined against purified CT using micro titration plates (Nunc, Denmark). ELISA will be made following the procedures of Nandy et al. [7] with slight modification. In brief, the micro titration plates were coated with 0.2 µg of purified CT (Sigma, St. Louis, USA) in PBS. Following 1 h incubation at 37°C, wells will be washed and blocked with 0.5% (w/v) bovine serum albumin (fraction V, Sigma). Next, wells will be filled with 100 µl of the test serum serially double diluted in PBS containing 0.5% BSA; the initial dilution will be 1:20 in all the samples. Following incubation and washing, 100 µl of appropriately diluted goat anti-human IgG peroxidase labeled conjugate (Sigma) will be added to each well and colour will be developed with the substrate solution *O*-phenylenedimine dihydrochloride (Sigma) and H_2O_2. Results will be recorded by measuring absorbance at 490 nm using an ELISA reader (BioRad, Hercules, USA) and titre will be expressed as the reciprocal of the highest dilution of antiserum that showed an OD_{490} value ≥0.200 in the assay.

Excretion of vaccine strain

Stools for two consecutive days collected from the participants will examined, graded and weighed. Faecal excretion of vaccine strain will be tested using conventional cultural and molecular methods. Stool specimens collected from the vaccine study participants will be collected in sterile containers and transported to the laboratory and processed within 2 hrs of collection. For qualitative analysis, the stool specimens are inoculated in alkaline peptone water (pH 8.0) and incubated for 6-8 hrs, followed by streaking on thiosulfate citrate bile salts sucrose (TCBS) agar (Eiken, Tokyo, Japan). For quantification of the vaccine strain, 1 gm of the stool specimen is serially diluted in sterile PBS and plated on to TCBS agar plates. Typical sucrose fermenting colonies are tested using biochemical tests and are serologically confirmed using antiserum specific for *V. cholerae* O1 Inaba. PCR assay is performed using previously published method [8] with all the isolated colonies targeting *ctxA* and *ctxB* genes for molecular confirmation of the vaccine strain.

4.3 Randomization & Blinding

4.3.1. Random Allocation

A randomization list will be prepared by a person not involved in the study either from NICED or from SAS. The randomization code will be sequential numbers unique to each individual. The single dose bottles containing either the vaccine or identical looking placebo will be arranged according to the randomization code and serially numbered. The master randomization chart will be prepared by a competent person not involved in the study. Permuted blocks of random numbers of fixed length will be used to randomize volunteers to vaccine or placebo in a 1:1 ratio. Separate serial number schemes for adults will be used.

4.3.2. Packaging and Coding

The vaccine will be packaged in single dose vials in a lyophilized form. Placebo will be packaged in single dose vials in a lyophilized form.

a) A live oral vaccine **VA1.4** will be used. This vaccine is identical in all respects to VA1.3 except that the Ampicillin resistance marker is absent. The dose will be freshly prepared in a diluent buffer provided by the manufacturer (Shantha Biotechnics) and administered within 5 minutes of preparing it, after administering a buffer solution as stated earlier.
b) Placebo containing lyophilized excepients including an appropriate amount of methyl cellulose of pharmaceutical grade to make it look identical with the vaccine containing bottles, will be mixed with the same diluent buffer as for the vaccine and administered within 5 minutes of reconstituting.
c) Serially numbered bottles single dose of either vaccine or a placebo will be administered to volunteers with the same serial numbers. For adults the serial number will start from 001.

4.4. Preparation of Live Oral Vaccine VA1.4
Shantha Biotechnics Pvt. Ltd. is contracted by DBT to prepare single dose lyophilized vaccine vial for use in this trial. They will also produce identical placebo single dose vials so that they look alike both in lyophilized form and after reconstitution with the diluent buffer.

4.5. Investigational Product Accountability
Shantha Biotechnics Pvt. Ltd. assisted by DBT and NICED will obtain the necessary clearances from the Drug Controller General of India (DCGI) for use in clinical trials of the study agents (cholera vaccine and placebo). The agents will be kept in a secure place. The investigator or the person in charge of the product management will maintain records of the product delivery to the trial site, the inventory at the site, the dose given to each subject, and the return of unused doses to Shantha Biotechnics Pvt. Ltd.

4.6 Storage conditions
The vaccine and placebo will be stored at 4°-8°C in a designated refrigerator kept close to the study unit to meet the daily needs.

4.7. Expected duration of follow-up of each subject is 21 days.

4.8. Premature Termination
Each subject is free to accept or reject the proposal to enroll himself/herself. Even after enrolment the subject will be able to withdraw from the study at any time.

4.9. Randomization Codes
The DBT will hold the code. The only persons in the field site with access to the codes will be with a designated member of DSMB and with the onsite clinical monitor in Kolkata, who will be handed the codes in sealed envelopes. They will be allowed only to un-blind codes in the event of severe putative vaccine reactions. Otherwise the codes will not be revealed until the end of the trial and follow-up and until the computerized data set to be used for the analysis of vaccine effectiveness has been frozen.

4.10. Identification of Source Data
The CRF forms will have the identification number only. Name of the volunteers will be on record only on the consent forms to be kept in a separate file. General instructions for filling the case report forms will be provided with CRF's.

4.11. Sample Size
A 1 –sided 95%confidence interval was used to calculate the sample size since this allowed us to rule out clinically unacceptable high rates of diarrheal adverse event occurring during the 3 days after either dose as well as establish that the vaccine induced adequate seroconversion to V. cholerae O1 Inaba among recipients [11].

Assuming a 10% diarrheal rate among placebo and vaccine recipients alike, to exclude a vaccine-placebo difference in the rate of diarrhea of greater than 20% (upper boundary of the 1-tailed 95% confidence interval) with a power of 0.9, the minimum number of subjects required for each group is 39. For serum vibriocidal responses, assuming a background rate of 5% seroconversion among placebo recipients after one dose and a true vibriocidal response in the vaccine group of 60%, to exclude a vaccine-placebo difference of 30% (lower boundary of the 1-tailed 95% confidence interval) with a power of 0.9, the minimum of subjects required for each group was 40. To adjust for the number of persons expected to drop out of the study, at least 44 persons are therefore required in each group.

4.12. Interpretation of the results:
(a) Acceptable level of adverse events mainly diarrhea, vomiting (see 4.11) is a necessary condition for field trial.
(b) Similar rise in vibriocidal antibody titre (8) as after VA 1.3 is necessary for going into a field trial (same or above the lower 95% CI confidence interval i.e. 49%).

Vibriocidal antibody is not regarded as a protective antibody against cholera, rather a surrogate marker of protection. It has been surmised from field studies in cholera endemic population where higher vibriocidal antibody titre (presumably from inapparent infection or mild disease due to cholera) is associated with less likelihood of clinical cholera. Based on these observations vibriocidal antibody titre has been used as a crude proxy for protection after administration of a candidate vaccine. However comparing a killed oral vaccine with a live oral vaccine using this proxy indicator for protection is likely to be misleading. One important difference is the ability of the live vaccine to colonize the intestine albeit for a brief period with relevant biological and immunological responses that may not be reflected in vibriocidal antibody rise.

5. SELECTION AND WITHDRAWAL OF SUBJECTS

5.1. Eligibility Criteria

Healthy adult men and non-pregnant women (aged 18 to 60 years) will be recruited from Kolkata for the trials. The process of obtaining consent, assessing eligibility, bleeding, and administering the vaccine and placebo, will occur in sequential steps during one session.

Recruitment

Residents of Kolkata not residing in the areas covered by the phase III trial or any other trial conducted by NICED or SAS where oral cholera vaccines were used will be recruited in the study. Recruitment will be performed in the SAS Clinical Trial Centre. Subjects will be instructed to proceed to the study center if they are interested in participating. Also it will be made sure that the subjects are recruited from the areas where no clinical trial is currently being performed. Key informants among the appropriate municipal wards will be contacted and the nature and purpose of the study explained for procuring volunteers

Inclusion Criteria

Healthy, non-pregnant adults aged 18 years to 60 years will be recruited in Kolkata.
All subjects must satisfy the following criteria at study entry:

1. Males or non-pregnant females aged 18 years and above who the investigator believes will comply with the requirements of the protocol (i.e. available for follow-up visits and specimen collection).
2. Written informed consent obtained from the subjects.
3. Healthy subjects as determined by:
    - Medical history
    - Physical examination and Lab tests
    - Clinical judgment of the investigator

Exclusion Criteria

The following criteria should be checked at the time of study entry, if any of the following is present then the subject will be excluded from the study:

1. Ongoing serious chronic disease
2. For females of reproductive age: Pregnancy (or females planning to become pregnant during the study period; as determined by verbal screening)
3. Immunocompromising condition or therapy (for corticosteroids this would mean ≥0.5 mg/kg/day) through verbal screening and history of recent ARI, diabetes, repeated attacks of diarrhoea
4. Diarrhea (3 or more loose/watery stools within a 24-hour period) 6 weeks prior to enrollment
5. One or two episodes of diarrhea lasting for more than 2 weeks in the past 6 months
6. One or two episodes of abdominal pain lasting for more than 2 weeks in the past 6 months
7. Intake of any anti-diarrhea medicine in the past week
8. Abdominal pain or cramps, loss of appetite, nausea, general ill-feeling or vomiting in the past 24 hours
9. Acute disease one week prior to enrollment, with or without fever. Temperature ≥38ºC warrants deferral of the vaccination pending recovery of the subject

10. Receipt of immunoglobulin or any blood product during the past 3 months
11. Receipt of antibiotics in past 14 days
12. Receipt of live or killed enteric vaccine in past 4 weeks
13. Receipt of killed oral cholera vaccine
14. Subject belonging to a population/family at high risk for HIV/AIDS such as migrant workers, commercial vehicle drivers, parenteral drug users, sex workers. Also in case of doubt the subject will be excluded.

Subject withdrawal during the study

Each subject is free to accept or reject the proposal to enroll in this study. Even after enrollment the subject will be able to withdraw from the study at any time.

The following criteria should be checked at each visit subsequent to the intake of the study agent:

1. Use of any immunosuppressive or immune-modifying drugs during the study period (for corticosteroids this would mean ≥0.5 mg/kg/day)
2. Administration of immunoglobulin or any blood product during the study period

If any of the above criteria is applicable, then it may affect the subject's evaluation in the per-protocol analysis, however the individual will not be withdrawn from the study.

The trial may have temporary moratorium if following vaccination one or more severe adverse events (SAE) occurs; the principal investigator will have the authority to call a temporary moratorium on vaccination, so that DSMB can review the side-effect data.

6. TREATMENT OF SUBJECT

After getting informed consent and ascertainment of eligibility, consenting eligible subjects will be entered into the trial in the randomization sequence, as noted earlier.

During administration of the agents, necessary numbers of vials with consecutive serial numbers (starting with Serial No. 1) will be at hand. The agent to be administered will be determined by the subject serial number which shall be the same as the serial number on the vial. The subject will drink a buffer solution (bicarbonates 2.5g and ascorbic acid 1.65g in 100 ml). The freeze dried vaccine or placebo vials will be reconstituted in 1ml of diluents provided by the manufacturer and given directly into month to drink 5 minutes after drinking the buffer solution. This will be followed by water ad-libitum. The agent will be administered with a disposable syringe (without needle). At the time of the vaccine dose, information about vaccine administration will be entered in the CRF 1. This information will note the code (serial number) of the assigned agent; the success of administration as well as certain additional information. If it is judged that a dose is not successfully ingested (e.g. regurgitated, spat out) recipients will be offered a replacement dose as per the discretion of PI.

The subject will not receive any other medicine unless indicated and a Medication Form will be filled. Study Doctor will decide about the medication; however antibiotics will be avoided if practical until they receive a single dose of Doxicycline or Azithromycin on day 21.

7. ASSESSMENT OF EFFICACY

7. 1. For serum vibriocidal responses (defined as ≥4 fold increase either at day 7 or 8) in ≥30% of the vaccine recipients will be considered as the vaccine having acceptable level of efficacy. The paired sera will be tested after completion of recruitment and follow-up.

7.2. For diarrheal adverse events, a rate lower than 10% incremental rate over the placebo group will be considered acceptable level of response. Analysis of adverse diarrheal events will be analyzed after completion of the study.

8. SAFETY

8.1. Adverse Events

Definitions

ADVERSE EVENTS:

An adverse event is defined as any noxious, pathologic, or unintended change in anatomic, physiologic, or metabolic functions, as indicated by physical signs, symptoms, and/or laboratory changes occurring in any phase of the clinical trial, regardless of their relationship to study medication. Adverse events include:

- an exacerbation of a pre-existing condition
- any illness happening in between the two doses' administration period
- any drug interaction
- any event related to a concomitant medication
- pregnancy

A treatment-emergent event is defined as any event not present prior to exposure to study medication or any event already present that worsens in either intensity or frequency following exposure to study medication.

Expected adverse events
Possible adverse events would include:

1) abdominal pain
2) loss of appetite
3) nausea
4) general ill feeling
5) fever and vomiting

Follow-up for adverse events following immunization will be conducted and recorded.

SERIOUS ADVERSE EVENTS:

A Serious Adverse Event means any event that results in:

- death
- is immediately life-threatening
- results in persistent or significant disability/incapacity
- requires inpatient hospitalization or prolongation of existing hospitalization
- Any other medically important condition that required intervention to prevent one of the above criteria.

Important medical events that may not result in death, be life-threatening, or require hospitalization may be considered a serious adverse event when, based upon appropriate medical judgment, they may jeopardize the subject and may require medical or surgical intervention to prevent one of the outcomes listed in this definition. Examples of such medical events include allergic bronchospasm requiring intensive treatment in an emergency room or at home, blood dyscrasias or convulsions that do not result in in-subject hospitalization, or the development of drug dependency or drug abuse.

Assessment of Causality

The Investigator's assessment of an adverse event's relationship to study drug is part of the documentation process, but it is not a factor in determining what is or is not reported in the study. If there is any doubt as to whether a clinical observation is a treatment-emergent adverse event, the event should be reported. The relationship of administration of the study agent to the serious adverse event will be assessed as follows:

Very Likely/Certain: A clinical event with a plausible time relationship to vaccine administration and which cannot be explained by concurrent disease or other drugs or chemicals.

Probable: A clinical event with a reasonable time relationship to vaccine administration; is unlikely to be attributed to concurrent disease or other drugs or chemicals.

Possible: A clinical event with a reasonable time relationship to vaccine administration, but which could also be explained by concurrent disease or other drugs or chemicals.

Unlikely: A clinical event whose time relationship to vaccine administration makes a causal connection improbable, but which could be plausibly explained by underlying disease or other drugs or chemicals.

Unrelated: A clinical event with an incompatible time relationship and which could be explained by underlying disease or other drugs or chemicals.

Unclassifiable: A clinical event with insufficient information to permit assessment and identification of the cause.

Assessment of Severity

The intensity of the adverse event will be rated adapting the guidelines, where applicable, set by the U.S. FDA Toxicity Grading Scale for Healthy Adult and Adolescent Volunteers Enrolled in Preventive Vaccine Clinical Trials [23] except for mild diarrhea, which will be defined as 3 loose or liquid stools in a 24 hour period.

**Systemic (General)**	**Mild (Grade 1)**	**Moderate (Grade 2)**	**Severe (Grade 3)**	**Potentially Life Threatening (Grade 4)**
Nausea/vomiting	No interference with activity or 1 - 2 episodes/24 hours	Some interference with activity or > 2 episodes/24 hours	Prevents daily activity, requires outpatient IV hydration	ER visit or hospitalization for hypotensive shock
Diarrhea	3 loose stools or < 400 gms/24 hours	4 - 5 stools or 400 - 800 gms/24 hours	6 or more watery stools or > 800gms/24 hours or requires outpatient IV hydration	ER visit or hospitalization

Headache	No interference with activity	Repeated use of non-narcotic pain reliever > 24 hours or some interference with activity	Significant; any use of narcotic pain reliever or prevents daily activity	ER visit or hospitalization
Fatigue	No interference with activity	Some interference with activity	Significant; prevents daily activity	ER visit or hospitalization
Illness or clinical adverse event (as defined according to applicable regulations)	No interference with activity	Some interference with activity not requiring medical intervention	Prevents daily activity and requires medical intervention	ER visit or hospitalization

Changes in the severity of an adverse event should be documented to allow an assessment of the duration of the event at each level of intensity to be performed. Adverse events characterized as intermittent require documentation of onset and duration of each episode.

8.2. Reporting of Serious Adverse Events (SAE)

SAEs will be reported promptly to the clinical monitor once the investigator determines that the event meets the protocol definition of a SAE. The SAE form will always be completed as thoroughly as possible with all available details of the event, signed by the investigator (or designee) and countersigned by the Clinical Trial Monitor. The investigator will always provide an assessment of causality at the time of the initial report. If deemed necessary by one or more SAEs, the monitor will have the authority to call a temporary moratorium on vaccination, so that get time to review the side-effect data.

8.3. Follow-up

Each subject will be followed up till 21 days of vaccination or till recovered from a serious adverse event if any.

9. STATISTICS

9.1. Analysis of Demographics

Demographic characteristics of subjects enrolled will be tabulated by group and overall.

Analysis of Safety
The number and percentage of subjects (with 95% CI) with at least one adverse event (solicited and/or unsolicited) after vaccination and during the 8 days and 21 days follow up period will be compared between the study groups.

The number and percentage of subjects with at least one Serious Adverse Event, with the frequencies of each type of event will be compared between the study groups.

Analysis of immunogenicity
Demonstration of at least a fourfold rise in serum anti O1 vibriocidal antibody titer will be the measure of vaccine immunogenicity using microtiter method with modification (Mahalanabis *et al.* 2009). The number and percentage of adults (with 95% CI) who exhibit at least a fourfold rise in serum anti-O1 vibriocidal titer after vaccination will be compared between the study groups.

The geometric mean titre among the vaccine and placebo groups at baseline and at day 7 or 8 and at 21 days will be compared. Geometric mean reciprocal folds rise in titre over 7-8 days and 21 days will also be compared between the vaccine and placebo groups. The proportion with ≥4 fold rise in titre (95% CI) will be compared using Binomial Exact method. The rate ratios (95% CI) for seroconversion between vaccine and placebo groups will also be calculated for 2×2 tables.

9.2. The level of significance for statistical tests will be 0.05.

9.3. Consideration for study termination are given under 5.2

9.4. Source data Form will be preserved securely locked using standard precautions by the Principal Investigators. Computerized data will have backup copies independently kept by the Principal Investigators and by the DBT. Xerox copies of the source data will also be retained by the Director, NICED securely locked.

9.5. We do not anticipate any deviation from the above statistical plan.

9.6. Analysis Plans

Both intention-to-treat and per-protocol analysis results will be described in the report.

Intention-to-Treat Analysis (ITT)
Every subject randomized in the study (who receive the correct or incorrect study agent, and complete or incomplete doses) will be analyzed, except if he/she did not receive any dose of the study agent (vaccine or placebo) or if no post randomization data collected for this subject.

Per-Protocol Analysis
A per protocol analysis will compare subjects according to the study agent actually received and will include only those subjects who satisfied the inclusion/exclusion criteria, followed the protocol, and received complete and correct doses. The following non-compliant subjects will be excluded:

- Subjects included without meeting at least one inclusion criterion.
- Subjects included despite meeting at least one exclusion criterion.
- Subjects found non compliant with the blood sampling schedule.
- Subjects vaccinated with wrong study agent (non compliance with the randomization code).
- Subjects excluded from the ITT analysis.

10. ACCESS TO SOURCE DATA/DOCUMENTS

It is hereby agreed that the Principal Investigators will permit trial-related monitoring, audit, IRB review, and regulatory inspections as may be needed and relevant by providing direct access to source data and documents.

11. QUALITY CONTROL AND QUALITY ASSURANCE

Clinical product quality control will be assured by Shantha Biotechnics Pvt. Ltd. DSMB will provide assurance on data safety and monitoring. Data records and documentation will have independent audit. On site clinical monitor will examine adverse event and serious adverse event forms.

12. ETHICS

The study will be performed in accordance with the ICMR Ethical Guidelines.

Risk-benefit: An identical construct of this vaccine has been tested in 304 subjects from the same area of the country and has been found to be nearly free from adverse effect. The potential benefits to the participants are substantial since cholera is endemic in Kolkata, India and a vaccine-preventive effect would translate into a real reduction of risk.

Justification for use of placebo in this study is that, no cholera vaccine is currently recommended for use in **Public Health Programs** in India, and a valid assessment of vaccine safety and immunogenicity in cholera endemic area is assured by a placebo group. A single dose vaccine has an added advantage especially in outbreak situations. The study will be implemented only after clearance from the Scientific Advisory Committee (SAC) and Institutional Ethics Committee (IEC) of NICED and the Ethics Review Committee of Society for Applied Studies.

13. DATA HANDLING AND RECORD KEEPING

13.1. Data entry

The team will manually check the filled forms for missing information, and data range and consistency of data on the day the forms are completed. Two data entry clerks will independently enter the data in databases created in Epi Info Software. The data files from the two clerks will be validated against each other for inconsistencies to detect any data transfer errors. These will be corrected after confirmation from the filled data forms. Finally, cleaned data will be merged with the main database at specified intervals and two backups taken, on a CD and External Hard Disk.

13.2. Data queries and error checks

Range and consistency checks will be inbuilt into the data entry system. The forms with data errors will be sent back to the study team for clarifications or corrections. The paper CRFs and CD backups will be stored in a separate building to avoid loss of data in the unlikely event of fire breaking out in the office. Fire proof steel cabinets will be used for storage.

13.3. Records to be kept

Participants will not be identified by name on any study documents except the permission forms. Case Report Forms (CRF) will have only the participant number, initials and the name of the study. The CRFs will serve as source documents. All data on the paper CRFs will be legibly recorded in blue ink (using ball point pen). The data will be directly entered into the computer from these CRFS. Any correction in the CRF will be made striking through the incorrect entry with single horizontal line and entering the correct information adjacent to it. The correction will be initialed and dated by the investigator or a study supervisor. Any requested information that is not obtained as specified in the protocol will have an explanation noted on the CRF as to why the required information was not obtained.

These paper CRFs will be filed in separate folders for each participant and stored under lock and key in the data management office after data entry.

The permission forms linking the name, addresses etc. with the participant numbers will be stored separately under lock and key in a separate office and will be accessible only to the principal investigators. The contact information is kept in a locked cupboard at the study office when the study is ongoing, and shifted to the locked cupboard holding the regulatory files once the study is completed.

13.4. Monitoring visits

Monitors will be sent from DBT as per sponsor guidelines.

13.5. Subject confidentiality

All records with the participant identification information will be kept in a locked cupboard in the principal investigators office and will be accessible to only the principal investigator and the co-investigators. All CRFs will only have subject Ids and initials and no other identifiers. Data will be provided only to the SAC and IEC if deemed necessary by them.

14. FINANCING AND INSURANCE

It is understood that the financing and insurance will be assured by DBT.

15. PUBLICATION

SAS, NICED and DBT will jointly own the rights to the data, specimen, results and other findings resulting from this study. The investigators will be entitled to publish the results in a collaborative fashion for the benefit of the public, taking care to protect the intellectual property rights to proprietary discoveries, if applicable.

16. REFERENCE LIST

1. Albert MJ, Qadri F, Wahed MA, Ahmed T, Rahman AS, Ahmed F, Bhuiyan NA, Zaman K, Baqui AH, Clemens JD, Black RE. Supplementation with zinc, but not vitamin A, improves seroconversion to vibriocidal antibody in children given an oral cholera vaccine. J Infect Dis. 2003 Mar 15;187 (6):909-13. Epub 2003.
2. Cholera Vaccines, Weekly Epidemiological Record. 2001; 76-117-124.
3. Clemens , J.D., F. Van Loon, D. A. Sack, J. Chakraborty, M.R. Rao, E. Ahmed, J.R. Harris, M.R. Khan, M. Yunus, and S. Huda. Field trial of oral cholera vaccines in Bangladesh: serum vibriocidal and antitoxic antibodies as markers of the risk of cholera. J. Infect Dis. 1991; 163:1235-1242.
4. Glass RI, A.M. Svennerholm, M.R. Khan, S. Huda, M.I. Huq and J Holmgren. Seroepidemiological studies of El Tor cholera in Bangladesh: associationof serum antibody levels with protection. I Infect Dis. 1985; 151: 236-242.
5. Levine MM and Kaper JB. Live Vaccines against cholera: an update. Vaccine 1993; 11: 207-212.
6. Levine MM and Pierce NF. Immunity and Vaccine Development, 1992; p. 285-327. In WB Greenough III and D. Barua (ed). Cholera. Plenum Press, New York.
7. Levine MM, JB Kaper, D. Herrington, J Ketley, GA Losomsky, CO Tacket, BD Tall and S.J. Cryz. Safety, immunogenicity and efficacy of recombinant live oral cholera vaccine CVD 103 and CVC 103-HgR. Lancet 1988; ii: 467-470.
8. Mahalanabis D, Ramamurthy T, Nair GB, Ghosh A, Shaikh A, Sen B, Thungapathra M, Ghosh RK, Pazhani GP, Nandy RK, Jana S, Bhattacharya SK. Randomized placebo controlled human volunteer trial of a live oral cholera vaccine VA1.3 for safety and immune response. Vaccine 2009;27:4850-4856
9. Migasena S, P. Pitisuttithan, B Prayurabong, P Suntharasami, W. Supanaranond, V. Desakorn, U Vongsthongsri, B. Tall, J. Ketley, G Losonsky, S. Cryz, J.B. Kaper and M.M. Levine. Preliminary assessment of the safety and immunogenicity of live oral cholera vaccine strain CVC 103-HgR in healthy Thai adults. Infect Immun 1989; 57: 3261-3264.
10. Mosley WH, McCormack WM, Ahmed A, Chowdhury AK, Barui RK. Report of the 1966-67 cholera vaccine field trial in rural East Pakistan. 2. Results of the serological surveys in the study population--the relationship of case rate to antibody titre and an estimate of the inapparent infection rate with Vibrio cholerae. Bull World Health Organ. 1969; 40(2):187-97.
11. Mosley WH, Ahmad S, Benenson AS and Ahmed A. The relationship of vibriocidal antibody titre to susceptibility to cholera in family contacts of cholera patients. Bull W.H.O. 1968a; 38: 777-785.
12. Mosley WH, Benenson AS and Barui RK. A serological survey for cholera antibodies in rural East Pakistan. I. The distribution of antibody in the control population of a cholera vaccine field-trial area and the relation of antibody titer to the pattern of endemic cholera. Bull W.H.O. 1968b; 38: 327-334.
13. Niyogi SK, Mondal S, Sarkar BL, Garg S, Banerjee D, Dey GN. Outbreak of cholera due to *V. cholerae* O1 in Orissa state. Indian J Med Res 1994; 100:217-8.
14. Qadri F, Mohi G, Hossain J, Azim T, Khan AM, Salam MA, Sack RB, Albert MJ, Svennerholm AM.Comparison of the vibriocidal antibody response in cholera due to Vibrio cholerae O139 Bengal with the response in cholera due to Vibrio cholerae O1. Clin Diagn Lab Immunol. 1995 Nov; 2(6):685-8.
15. Radhakutty G, Sircar BK, Mondal SK, Mukhopadhyay AK, Mitra RK, Basu A, Ichhpujani RL, Nair GB, Bhattacharya SK. Investigation of the outbreak of cholera in Alleppey & Palghat districts, south India. Indian J Med Res. 1997 Nov; 106:455-7
16. Sanchez JL, Trofa AF, Taylor DN, Kuschner RA, DeFraites RF, Craig SC, Rao MR, Clemens JD, Svennerholm AM, Sadoff JC, et al. Safety and immunogenicity of the oral, whole cell/recombinant B subunit cholera vaccine in North American volunteers. J Infect Dis. 1993 Jun;167 (6):1446-9.
17. Su-Archawatana, P., P. Singharaj, D. Taylor, C. Hoge, A. Trofa, K. Kuvanont, S. Migasena, P. Pitisuttitham, Y.L. Lim, G. Losonsky, JB Kaper, SS Wasserman, S. Cryz, P. Echeverria, and M.M. Levine. Safety and immunogenicity of different immunization regimens of CVC 103-HgR live oral cholera vaccine in soldiers and citilians in Thailand. J Infect Dis 1992; 165: 1042-1048.
18. Sur D, Dutta P, Nair GB, Bhattachary SK. Severe cholera outbreak following floods in a northern district of West Bengal. Indian J Med Res 2000; 112:178-80.

19. Thungapathra M, Sharma C, Gupta N, Ghosh RK, Mukhopadhyay A, Koley H, Nair GB, Ghosh A. Construction of a recombinant live oral vaccine from a non-toxigenic strain of Vibrio cholerae O1 serotype Inaba biotype E1 Tor and assessment of its reactogenicity and immunogenicity in the rabbit model. Immunol Lett. 1999 Jun 1; 68(2-3):219-27.
20. WHO Global Task Force on Cholera Control, Cholera Vaccine: A new public health tool? Report of a WHO meeting, 10-11 December 2002, Geneva, Switzerland.
21. World Health Organization Expert Committee on Biological Standardization. Guidelines for the production and control of inactivated oral cholera vaccines. 26-30 November 2001. Geneva. BS/01/1938.
22. Mahalanabis D, Lopez AL, Sur D, Deen J, Manna B, Kanungo S, Seidlein L, Carbis R, Han SH, Shin SH, Attridge S, Rao R, Holmgren J, Clemens J, Bhattacharya SK. A Randomized, Placebo-Controlled Trial of the Bivalent Killed, Whole-Cell, Oral Cholera Vaccine in Adults and Children in a Cholera Endemic Area in Kolkata, India. PloS One 2008; 3(6):e2323.
23. United States Food and Drugs Administration Center for Biologics Evaluation and Research. Toxicity Grading Scale for Healthy Adult and Adolescent Volunteers Enrolled in Preventive Vaccine Clinical Trials available at http://www.fda.gov/ cber/gdlns/toxvac.htm, accessed on 4 December 2007.

17. ANNEXURES
